# Artificial Nutrition Support During Acute Illness in Pregnancy: A Scoping Review

**DOI:** 10.1111/jhn.70315

**Published:** 2026-07-20

**Authors:** Danielle E. Bear, Hanna Tejani, Ali Morrison, Kevin Whelan

**Affiliations:** ^1^ Department of Nutritional Sciences King's College London London UK; ^2^ Department of Nutrition and Dietetics Guy's and St Thomas' NHS Foundation Trust London UK; ^3^ Department of Critical Care Guy's and St Thomas' NHS Foundation Trust London UK

**Keywords:** enteral nutrition, hospitalisation, parenteral nutrition, pregnancy

## Abstract

**Background:**

The delivery of artificial nutrition support during acute illness in patients who are pregnant is complex with considerations for both maternal and foetal health outcomes. However, there is little known about the research available to guide the artificial nutrition support during pregnancy. This scoping review aims to examine the literature related to artificial nutrition support practices during acute illness in pregnancy, to understand current research, identify gaps and inform directions for future research.

**Methods:**

The review was conducted in accordance with the Joanna Briggs Institute (JBI) methodology for scoping reviews. A systematic search of five databases (MEDLINE, Embase, CINAHL, Web of Science and the Maternal and Infant Care Database (MIDIRS)) was conducted. Eligible studies involved adult pregnant patients aged 18 or over, hospitalised across a variety of care settings, and receiving artificial nutrition in the form of enteral nutrition or parenteral nutrition. Studies not written in English Language were excluded. Data were extracted on weight, gestation period, type and route of feeding, indication for artificial feeding, energy target and provision, protein target and provision, duration of intervention, micronutrient delivery and supplementation, method of delivery, foetal birthweight and maternal and foetal outcomes.

**Results:**

A total of 24 studies were included of which the majority were case reports (*n* = 12 studies), followed by case series (*n* = 7), and only one randomised controlled trial (early enteral feeding vs standard care). The most common indication for artificial nutrition support was hyperemesis gravidarum (*n* = 14 studies) and the most common method was parenteral nutrition (*n* = 14). There was considerable heterogeneity in reporting of energy and protein targets and delivery, micronutrient supplementation, weight change over pregnancy and maternal and foetal outcomes.

**Conclusions:**

This scoping review highlighted the very limited number of studies regarding artificial nutrition support during pregnancy. Hugh quality research is urgently required to inform guidelines and standardise and optimise management strategies and improve outcomes in this complex patient cohort.

## Summary

- Evidence for artificial nutrition support during acute illness in pregnancy is extremely limited, resulting in heterogeneous practice and reliance on individual clinical judgement.
- Energy and protein requirements are not validated for this population, requiring pragmatic estimation, and frequent reassessment of nutritional adequacy.
- Micronutrient provision and nutritional monitoring are inconsistently reported, highlighting the need for proactive supplementation and alternative anthropometric measures when weight is unavailable.

## 1 Introduction

Nutrition plays a significant role in healthy pregnancies from pre‐conception to post‐partum, with a plethora of research supporting its role in both maternal and foetal outcomes [1]. Specifically, the early life theory highlights the importance of the ‘first 1000 days’, and the central role of nutrition in infant and child development [2]. However, pregnancy is a metabolically and clinically challenging period and maternal and foetal complications may lead to unscheduled attendances to hospital for obstetric or medical emergencies. One of the most common reasons for hospital admissions is hyperemesis gravidarum (HG) [3], a severe form of persistent nausea and vomiting, resulting in weight loss, dehydration and electrolyte imbalance [4]. A population‐based cohort study in England of 8.2 million pregnancies, found hospital admissions due to HG alone in 1.75% pregnancies [5]. Furthermore, the National Maternal and Perinatal Audit, highlighted the rate of admissions of pregnant patients to the Intensive Care Unit (ICU) was 2.4 per 1000 pregnancies (0.24% of pregnancies) [6]. Common reasons for admission to ICU include hypertensive disorders such as preeclampsia, obstetric haemorrhage, sepsis and anaesthetic complications [7]. Across these varied presentations, acute illness during pregnancy significantly elevates nutritional risk. Persistent vomiting, prolonged fasting, increased metabolic demands and reduced oral intake may each necessitate the use of artificial nutrition support, either enteral nutrition (EN) or parenteral nutrition (PN), to maintain adequate nutritional status for both mother and foetus.

Despite this range of clinical conditions during pregnancy, the existing literature has focussed primarily on the role of nutrition during “healthy” pregnancy [8] which is problematic for those who are acutely ill, pregnant and admitted to hospital. Specifically, the use of artificial nutrition support in acutely ill patients who are pregnant can present numerous nutritional challenges, for which evidence largely relates to pregnant patients who are not acutely ill. For example, there is no synthesis of research in relation to the energy and protein costs of pregnancy specifically during illness, the most accurate methods of estimating energy and protein requirements, or micronutrient supplementation (including folate supplementation), and optimal feeding routes during acute illness. It is therefore unclear how nutrition management should differ during pregnancy with and without acute illness, especially those requiring artificial nutrition support such as enteral nutrition (EN) or parenteral nutrition (PN). The lack of evidence directing care of the acutely unwell pregnant patient requiring artificial nutrition support may result in wide variation in care with an unknown impact on maternal and foetal outcomes.

Therefore, the aim of this scoping review is to examine the published literature related to artificial nutrition support practices during acute illness in pregnancy, with a specific focus on [1]: common indications for artificial nutrition support [2]: the type and route of artificial nutrition support used [3]; energy and protein prescription targets and delivery [4]; weight change across gestation; and [5] reported maternal and foetal outcomes in order to identify current evidence, highlight gaps in practice and reporting, and inform directions for future research.

## 2 Material and Methods

### 2.1 Protocol and Registration

This scoping review was conducted in accordance with the Joanna Briggs Institute (JBI) Manual for Evidence Synthesis [9] and reported as per Preferred Reporting Items for Systematic Reviews and Meta‐Analyses Extension for Scoping Reviews (PRISMA‐ScR) guidelines [10]. The protocol was pre‐registered under the Open Science Framework [11].

### 2.2 Search Strategy

A systematic search of the scientific literature was conducted in March 2024 across five databases (MEDLINE, Embase, CINAHL, Web of Science and the Maternal and Infant Care Database (MIDIRS)) with no date restriction applied. The search strategy was developed with the support of a librarian. Studies were restricted to the English language. Search alerts were set up to capture any further updates in the literature during the period of the review. A full list of search terms are available in supplementary Appendix S1. The ClinicalTrials.gov clinical trials registry was searched for completed, unpublished studies. Backwards citation searching was undertaken by screening the reference lists of eligible full text articles.

### 2.3 Study Eligibility

Sources of evidence were included if they met the eligibility criteria outlined in Table 1.

**Table 1:** Population, concept and context (PCC) and type of evidence inclusion.

Criteria	Inclusion	Exclusion
Population	Adult pregnant patients aged 18 or above and hospitalised across a variety of care settings whilst receiving artificial nutrition, as sole enteral or parenteral nutrition or as supplement to oral intake. While this scoping review uses terms such as “mother,” “maternal” to reflect the language used in the included literature, the authors recognise that not all people who experience pregnancy identify as female or as mothers. This review intended to be inclusive of all individuals capable of becoming pregnant, including transgender men and people who are non‐binary, and studies involving any such individuals would have been eligible for inclusion.	
Concept	Studies that investigated the use of enteral nutrition or parenteral nutrition during pregnancy and acute illness.	Studies focused on oral intake only. Where studies reported mixed populations on either oral or artificial nutrition, studies were excluded if data on those receiving artificial nutrition could not be extracted.
Context	Studies conducted in the hospital setting, including the intensive care unit, with no limit to the duration of admission. Evidence sources included RCTs, experimental and observational studies, case reports, case series, and conference abstracts.	Reviews, opinion papers or any reports without original data were excluded. Animal studies were not eligible.

### 2.4 Screening and Selection

Search results were exported and uploaded to Covidence (Veritas Health Innovation, Melbourne, Australia), where duplicates were identified and removed automatically. The search results were screened in duplicate independently by two researchers (HT and AM). Abstracts and titles were screened as per the eligibility criteria, and disagreements were discussed by the two researchers until agreement was reached. In the case where there was disagreement, a third independent researcher (DEB) arbitrated. Following this, full text articles were obtained and screened against the eligibility criteria in duplicate and independently by the same two researchers with any disagreements resolved by a third researcher (DEB).

### 2.5 Data Extraction, Analysis, and Presentation

A draft data extraction table was developed and piloted in Covidence (Veritas Health Innovation, Melbourne, Australia) and used by the two researchers to independently extract data from the full text literature, this was revised as necessary during the extraction period. Results of the data extraction from both researchers was cross checked and a consensus was made to form the final set of data for review.

Data extracted from all studies included citation details (author/s, date, journal, volume, issues and pages), country of origin, study design, population, aims and outcomes. Details in relation to the concept of this review included, weight, gestation period, type and route of feeding, indication, duration and method of artificial nutrition support, energy and protein target and provision, micronutrient delivery and supplementation, foetal birthweight and maternal and foetal outcomes (supporting information Appendix S2). Data was exported into Excel (Microsoft Corporation, Redmond, WA, USA) and descriptive analysis was undertaken.

## 3 Results

### 3.1 Search Results

**Figure 1:**
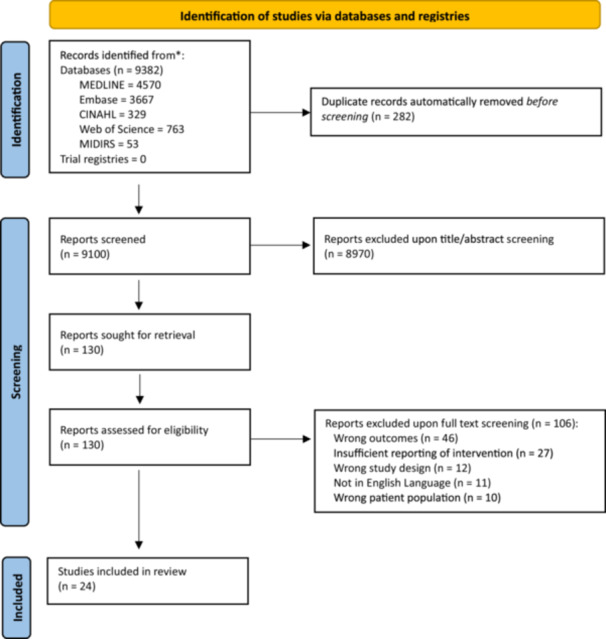
PRISMA‐ScR flow diagram illustrating studies identified, screened, included and excluded.

A search across databases yielded 9100 studies after 282 duplicates were removed as presented in the PRISMA flow diagram in Figure 1. Title and abstract screening resulted in 8970 records being excluded leaving 129 for full text screening. From these, 105 were excluded. The grey literature search yielded no papers leaving 24 papers available for data extraction and analysis.

### 3.2 Characteristics of Included Articles

#### 3.2.1 Study Characteristics

Characteristics of the included literature are summarised in Table 2. The 24 publications were published between 1982 and 2023, covering 41 years. There was geographic variation in populations, with 11 countries represented overall. The majority of studies were from the United States (*n* = 8) [16, 19, 20, 21, 22, 31, 33, 34]. Overall there were six study designs with 12 case reports [15, 16, 22, 23, 24, 27, 29, 32, 33, 34, 35, 36] being the most common, followed by 7 case series [12, 13, 14, 19, 20, 21, 31]. There was only one randomised controlled trial, which was an open label RCT investigating the effect of early EN plus standard care compared with standard care alone on birth weight in women with HG [18]. In relation to the interventions relevant to this scoping review HG was the most common indication (*n* = 15) for artificial nutrition support in this cohort [13, 14, 17, 18, 19, 21, 23, 25, 28, 29, 30, 31, 32, 35]. Other indications for artificial nutrition support included weight loss and inability to tolerate oral intake or EN. The duration of artificial nutrition support differed across the 24 studies, with interventions ranging from 1 day to 6 months.

**Table 2:** Summary of information on population, presentation, and summary of artificial nutrition support practices reported in the included studies (*n* = 24).

Author, year	Study design	Country	Population (number)	Presenting complaint to hospital	Artificial nutrition support		
					Type	Indication	Route
Adami et al, 1992 [12]	Case series	Italy	Post biliopancreatic diversion (*n* = 11)	Malnutrition marked by low serum albumin	Parenteral	Weight loss and reduced oral intake/Low serum albumin and small for gestational age foetus Low serum albumin and weight loss	Central venous catheter
Boyce, 1992 [13]	Case series	Australia	Hyperemesis gravidarum (*n* = 2)	Hyperemesis gravidarum and weight loss	Enteral	Weight loss and unable to tolerate oral intake	Nasogastric
Christodoulou et al, 2008 [14]	Case series	Greece	Hyperemesis gravidarum (*n* = 2)	Exacerbation of Hyperemesis gravidarum with intractable vomiting, dehydration and exhaustion	Parenteral	Nausea and persistent vomiting Esophagitis and erosive gastritis of the fundus	Peripheral
D'Orsi et al, 2023 [15]	Case report	Italy	Cystic Fibrosis (CF) (*n* = 1)	Four pulmonary infective exacerbations and gradual decrease of pulmonary function and need of non‐ invasive oxygen support	Parenteral	Deterioration of nutritional status (weight loss)	Not reported
Fedorka and Sullivan, 2004 [16]	Case report	United States	Trauma (*n* = 1)	Motor vehicle accident and re‐admitted with diabetes insipidus	Parenteral and enteral	Comatose state	PEG
Galetta et al, 2022 [17]	Cross Sectional	Brazil	Hyperemesis gravidarum (*n* = 26)	Hyperemesis gravidarum	Mixed	Weight lossLoss of appetite Nausea Vomiting	Not reported
Grooten et al, 2017 [18]	Randomised control trial	Netherlands	Hyperemesis gravidarum (*n* = 116)	Severe nausea	Enteral	Severe weight loss, electrolyte imbalance, severe dehydration	Nasogastric, or nasojejunal in 12% of enteral feeding group and 2% in standard care group
Hsu et al, 1996 [19]	Case series	United States	Hyperemesis gravidarum (*n* = 7)	Intractable nausea, vomiting, dehydration and weight loss	Enteral	HG and weight loss	Nasogastric
Laglenne and Mogensen, 2022 [20]	Case series	United States	Gastroenterology – mixed (*n* = 2)	Patient 1: Infarcted ileum with internal hernia within mesentery requiring small bowel resectionPatient 2: eclamptic seizure, ventricular tachycardia and small bowel resection	Patient 1 – Parenteral Patient 2 –Parenteral and enteral	Short bowel syndromeWeight loss and high volume diarrhoea	Patient 1 – PN Patient 2 – PN and PEG
Maxwell et al, 2010 [21]	Case series	United States	Mixed (*n* = 9)	5 = Hyperemesis gravidarum3 = gastroparesis1 = gastric outlet obstruction	Enteral	5 for HG, 3 for gastroparesis, 1 for gastric outlet obstruction	PEG = 6RIG = 1Jejunostomy = 2
Mulherin et al, 2019 [22]	Case report	United States	Cystic fibrosis (*n* = 1)	Cystic Fibrosis exacerbation and failure to thrive due to persistent nausea and vomiting	Parenteral	Poor EN tolerance and poor weight gain	Central venous catheter
O'Connor and Quinn, 1994 [23]	Case report	United Kingdom (Scotland)	Hyperemesis gravidarum (*n* = 1)	Severe vomiting, negative nitrogen	Parenteral	Severe vomiting, weight loss, unsuccessful nasogastric tube insertion	Tunnelled subclavian line
Okon and Hussein, 1996 [24]	Case report	United Kingdom	Caustic laryngeal stricture (*n* = 1)	1 week history of inability to swallow following ingestion of 98% sulphuric acid	Parenteral	Oesophagitis and distal oesophageal mucosal irregularity ‐ inability to swallow fluid or solids	Subclavian catheter
Peled et al, 2014 [25]	Retrospective observational	Israel	Hyperemesis gravidarum (*n* = 599)	Hyperemesis gravidarum	Parenteral	If clinical symptoms or laboratory testing did not resolve after administration of IV fluids and antiemetics or oral intake could not be restored within 72hrs, PN was warranted along with 10% loss of body weight from pre‐pregnancy weight	Not reported
Roem, 2002 [26]	Case report	Australia	Hyperemesis gravidarum (*n* = 1)	Four‐week history of hyperemesis and 3 kg weight loss	Enteral	A loss of 14% pregnancy weight following initiation of oral nutritional supplements and dietary advice	Nasogastric
Spiliopoulos, 2013 [27]	Case report	Not specified	Oesophageal achalasia (*n* = 1)	Occasional vomiting, dysphagia for liquids and solids, anorexia, mild uterine contractions and 9 kg weight loss over 4‐week period	Parenteral	Vomiting, dysphagia for liquids and solids Malnutrition indicated by 9 kg weight loss over 4 weeks	Peripheral line
Stokke et al, 2015 [28]	Cohort study	Norway	Hyperemesis gravidarum (*n* = 588)	Hyperemesis with dehydration, weight loss or ketonuria/electrolyte imbalance	Mixed	If food intake was not resumed within 2–3 days peripheral PN was commenced	Peripheral line for PN or nasojejunal for EN
Tanabe et al, 2022 [29]	Case report	Japan	Hyperemesis gravidarum (*n* = 1)	Severe Hyperemesis gravidarum	Parenteral	Lost 5 kg from pre pregnancy weight due to severe Hyperemesis gravidarumpoor oral intake	Peripheral line
Vaisman et al, 2004 [30]	Retrospective observational	Israel	Hyperemesis gravidarum (*n* = 11)	Severe vomiting, electrolyte abnormalities, persistent ketonuria and weight loss	Enteral	Vomiting and weight loss	Nasojejunal
Vanderwoude and Scholten, 1995 [31]	Case series	United States	Hyperemesis gravidarum (*n* = 6)	Hyperemesis gravidarum	Parenteral – with 3 patients discharged on home PN	HG and weight loss of average 8%	Central venous catheter
Webber and Field, 1993 [32]	Case report	United Kingdom	Hyperemesis gravidarum (*n* = 1)	1 week history of vomiting, mild dehydration, heavy glycosuria and ketonuria	Parenteral	Weight loss and evidence from foetal growth chartOesophagitis	Not reported
Weinberg et al, 1982 [33]	Case report	United States	Hyperlipidaemic pancreatitis (*n* = 1)	2‐day history of nausea and vomiting and severe epigastric pain	Parenteral	Attempted to commence oral diet but due to increasing plasma triglycerides and loss of 5.3 kg from admission weight followed by poor tolerance to enteral feeding tube	Not reported
Wessel et al, 2015 [34]	Case report	United States	Maple syrup urine disease (*n* = 1)	Management of maple syrup urine disease and presenting with mild nausea and vomiting	Supplementary parenteral	Increased BP and preeclampsia and to meet metabolic demands of underlying MSUD	PICC line
Zhao et al, 2017 [35]	Case report	China	Hyperemesis gravidarum (*n* = 1)	Persistent vomiting for 1 month with decreased urine volume and unable to eat for 5 days	Parenteral	Weight lossCould not tolerate food and ONSRefused nasogastric feeding	Peripheral venous

Abbreviations: BP, blood pressure; EN, enteral nutrition; IV, intravenous; KG, kilograms; ONS, oral nutritional supplements; PEG, percutaneous endoscopic gastrostomy; PICC, peripherally inserted central catheter; PN, parenteral nutrition; RIG, radiologically inserted gastrostomy.

### 3.3 Artificial Nutrition Support in Hyperemesis Gravidarum

Hyperemesis gravidarum was the most common indication for artificial nutrition support identified in this review (*n* = 15 studies). Across the HG studies, PN was the predominant form of nutrition support, although EN via nasogastric or nasojejunal tube was also used. The available evidence suggests that the threshold for initiating PN in HG is typically persistent vomiting with weight loss, electrolyte disturbance, or failure to tolerate EN. Only one RCT was identified in this population, the MOTHER trial [18], which found no significant difference in birth weight with early EN compared with standard care in HG. However, 70% of patients in the EN group reported at least one EN‐associated complication. In contrast, a retrospective observational study reporting that PN support in HG was associated with higher birth weight and lower rates of composite neonatal morbidity compared with controls [25], suggesting a potential benefit of timely nutritional intervention. Energy and protein prescription practices varied considerably across HG studies, with limited reporting of targets and no study reporting delivery relative to target. Despite HG being a prolonged condition with significant nutritional risk, the paucity of data on energy and protein adequacy in this group is a notable gap.

### 3.4 Artificial Nutrition Support in Cystic Fibrosis During Pregnancy

Two case reports described artificial nutrition support during pregnancy in patients with cystic fibrosis (CF) [15, 22], both using PN. One case report described long‐term PN use over 85 days in a patient with CF, with energy targets progressively increased from 1444 to 2790 kcal/day (up to 61 kcal/kg/day). Due to failure to achieve target weight gain, the infant was born small for gestational age [22]. The second case report similarly reported weight loss to below pre‐pregnancy levels and a late preterm delivery [15].

### 3.5 Other Indications for Artificial Nutrition Support

The remaining studies covered a range of varied and rare indications including trauma, maple syrup urine disease, short bowel syndrome, oesophageal achalasia, caustic laryngeal stricture, hyperlipidaemic pancreatitis, and gastroparesis. While these individual case reports and case series provide limited generalisable evidence, they collectively illustrate that artificial nutrition support may be required across a wide spectrum of acute illness during pregnancy. Reporting from these cases is useful for raising awareness of rare but important clinical scenarios and for informing pragmatic decision‐making when no specific guidance exists. However, the lessons regarding energy and protein targets, route selection, and monitoring discussed in this review the following sections apply across all these indications and should be read in the context of the underlying clinical condition driving nutritional support.

## 4 Nutrition Intervention and Monitoring

### 4.1 Energy and Protein Targets

The most common form of artificial nutrition support was PN (*n* = 14) [12, 14, 15, 16, 22, 23, 24, 25, 27, 29, 31, 32, 33, 34, 35], with four studies [16, 17, 20, 28], implementing mixed feeding methods with both EN and PN (Table 3).

**Table 3:** Summary of artificial nutrition support interventions in included studies.

Paper	Type of ANS	Energy delivery	Protein delivery	Micronutrient provision (amount)	Duration of ANS	Body weight			
						Pre‐pregnancy	Start of ANS	Last reported	Net change
Adami et al, 1992 [12]	Parenteral	800–2200 kcal ‐ (2 l/day including 250 g/day 50% dextrose and 10%–20% fat emulsion ‐ Intralipid) 1500 kcal–50 g/day of 20% intralipid Supplementary PN group 500–1300 kcal/day	85 g amino acids/day, supplementary PN group 42–85 g amino acids/day	Not reported	12 weeks 20 days and restarted for 30 days	Patient 1: 108 kg Patient 2: 83 kg	Patient 1: 90 kg Patient 2: 83 kg	Patient 1: Not reported Patient 2: 90 kg	Patient 1: ‐18 kg Patient 2: +7 kg
Boyce, 1992 [13]	Enteral	2400 kcal/day	90 g/day	Not reported	6–15 days	60 kg	53.9 kg	57.1 kg	+3.2 kg
Christodoulou et al, 2008 [14]	Parenteral	Clinomel N4‐555 (1215) enriched with 1 ampoule of glutamine	Not reported	Not reported	12–14 days	Not reported	Not reported	Not reported	Not reported
D'Orsi et al, 2023 [15]	Parenteral	Not reported	Not reported	Not reported	Not reported	Not reported	Not reported	54 kg	‐2 kg
Fedorka and Sullivan, 2004 [16]	Parenteral and enteral feeding	Osmolite 80 ml/hr Osmolite HN 80 ml/hr Osmolite 100 ml/hr on discharge	Not reported	Magnesium oxide (800 mg) Ferrous sulphate (225 mg) Multivitamin (amount not stated)	PEG inserted at 10th week of pregnancy and used continuously	Not reported	Not reported	Not reported	Not reported
Galetta et al, 2022 [17]	Mixed	2023 + /− 409.5 kcal/day	62.8 + /‐ 8.1 g	Not reported	Not reported	59.3 SD 7.3	59.7 SD 11.4	58.1 kg SD 6.6	‐1.7 kg +/‐ 2.3 kg
Grooten et al, 2017 [18]	Enteral	Feeding regimens were followed according to local protocol	Feeding regimens were followed according to local protocol	Not reported	Median duration = 7 daysIQR = 1–34 days	EN = 72.8 kg SD 5 SC = 69.8 kg SD 14.6	EN = 70.3 kg SD 16.6 SC = 65.5 kg SD 13.4	Not reported	EN = ‐2.5 kg SD 4.3S C = 4.3 kg SD 4.1
Hsu et al, 1996 [19]	Enteral	1540‐2570 kcal	Not reported	Not reported	5–174 days Mean = 43 days	Not reported	Not reported	Not reported	Not reported
Laglenne and Mogensen, 2022 [20]	Mixed	Not reported	Not reported	Daily complete multivitamin with minerals, vitamin D supplementation	2–3 months	Not reported	Patient 1: 95 kg Patient 2: 125 kg	Patient 1: 100.9 kg Patient 2: 114 kg	Patient 1: +5.9 kg Patient 2: ‐11 kg
Maxwell et al, 2010 [21]	Enteral	Not reported	Not reported	Not reported	Mean duration of 24.6 SD 12.1 weeks	Not reported	Not reported	Not reported	7.7 kg SD 5.6 kg average weight gain
Mulherin et al, 2019 [22]	Parenteral	1444 kcal/d Advanced to 2552 kcal/day Advanced to 2790 kcal/day Based on 61 kcal/kg/day	75 g amino acids across peripartum period	Not reported	85 days + 4 weeks post‐partum	40.3 kg	41 kg	46 kg at birth 41.8 kg post‐partum	+5 kg
O'Connor and Quinn, 1994 [23]	Parenteral	2200 kcal/day including 100 g lipid	Not reported	Not reported	8 weeks	Not reported	42.7 kg	44.5 kg	+1.8 kg
Okon and Hussein, 1996 [24]	Parenteral	2030–2550 kcal 1800–2200 kcal (non‐protein)	57–85 g/kg/day	Trace elements and vitaminsIron (100 mg)	Commenced at 28 weeks Continued post‐partum, unclear timeframe	Not reported	Not reported	Not reported	+7.3 kg since admission
Peled et al, 2014 [25]	Parenteral	Not reported	Not reported	Not reported	Not reported	Not reported	Not reported	Not reported	Not reported
Roem, 2002 [36]	Enteral	2100 kcals/day	74 g	Phosphate (1 g) Magnesium (0.69 mmol/L) Thiamine (3.2 mg)	5 weeks	51 kg	43 kg	52.5 kg	+9.5 kg
Spiliopoulos et al, 2013 [27]	Parenteral	1215 kcal/day	Not reported	Not reported	8 weeks	Not reported	62 kg	Not reported	+2 kg
Stokke et al, 2015 [28]	Mixed	Fresubin 20 ml/hr every 8 h and increased to 80 ml/hr = 2000 ml/24hrs = 2000 kcals	Not reported	Not reported	Median for tube feeding was 5 days. Up to 41 days	Not reported	Median (95% CI)IV fluid: 61 (59–62.5)Peripheral nutrition: 61 [58–63]Enteral nutrition: 60.5 [58–64]	Not reported	‐0. 5 kg
Tanabe et al, 2022 [29]	Parenteral	1040 kcal/day	Not reported	Zinc acetate dihydrate (150 mg/d)	4 weeks	5 0 kg	45 kg	44.3 kg	‐0.7 kg
Vaisman et al, 2004 [30]	Enteral	Osmolite 1 kcal/ml Initial rate = 40 ml/hr Increased until maximum rate = 100 ml/hr Provided over 24hrs	Not reported	Not reported	1–21 days	Not reported	47.1–83.1 kg	49.1–84.7 kg	Mean weight loss = 0.9‐5.1 kg (2.2 SD 1.1)
Vanderwoude and Scholten, 1995 [31]	Parenteral – with 3 patients discharged on home PN	40–50 kcal/kg/day	2‐3 g/kg/day	Daily vitamins and trace elements provided	7–58 days (mean = 19)	Not reported	Not reported	Not reported	3%–12% decrease in body weight. All had weight loss of at least 8 lbs.
Webber and Field, 1993 [32]	Parenteral	Not reported	Not reported	Not reported	8 weeks	Not reported	53 kg	58 kg	+5 kg
Weinberg et al, 1982 [33]	Parenteral	2400 kcals/day	100 g/day	Not reported	2 weeks	Not reported	50.7 kg	56.4 kg	+5.7 kg
Wessel et al, 2015 [34]	Supplementary parenteral	2500 kcals	70 g protein of BCAA free protein and added leucine, isoleucine and valine	Sodium chloride (43 mEq) Potassium chloride (63 mEq) Magnesium sulphate (8 mEq) Calcium gluconate (10 mEq) Sodium phosphate (15 mmol) Multiple trace element (1 ml)	From delivery to 10 days postpartum	70.5 kg	Not reported	Not reported	+13 kg weight gain over pregnancy
Zhao et al, 2017 [35]	Parenteral	150 g glucose, 4.5 g sodium chloride, 50 g MCT/LCT fat emulsion	42.5 g amino acids	Potassium chloride (4.5 g) Calcium gluconate (1 g) Magnesium sulphate (1 g) Soluvit (1 vial) Vitalipid (1 vial) Addamel (1 vial) Sodium glycerophosphate (2.16 g)	12 days	69.5 kg	66 kg	63 kg	‐3 kg

Abbreviations: ANS, artificial nutrition support; BCAA, branched chain amino acids; EN, enteral nutrition; IQR, interquartile range; iU, international unit; KG, kilograms; LBS, pounds; LCT, long chain triglyceride; MCT, medium chain triglyceride; mEq, milliequivalent; PEG, percutaneous endoscopic gastrostomy; PN, parenteral nutrition; SC, standard care; SD, standard deviation.

Energy targets, and the methods used to calculate them, were detailed in only six studies [17, 19, 22, 23, 30, 34] with one [18] stating only that targets were estimated as per local policy. Two studies [22, 34] estimated energy requirements at 35 kcal/kg/d, with one advancing the provision from 35 kcal/kg/d to 55 kcal/kg/d and 61 kcal/kg/d [22] due to failure to meet goal weight. One of these studies did not specify the basis for the weight used in the equation (18) and the other used pre‐gravid weight [34]. Two studies used predictive equations to determine energy requirements using the Harris‐Benedict equation (17) and Institute of Medicine (IOM) formula for energy requirements [17]. Both studies adjusted for pregnancy with an additional 300 kcal/d added to the Harris Benedict equation and 0 kcal/d, 340 kcal/d, and 452 kcal/d across the first, second and third trimester respectively to the IOM formula.

Energy delivery was reported in 18 studies and ranged from 1040 kcal/d to 2790 kcal/d [12, 14, 16, 17, 19, 22, 23, 24, 27, 28, 29, 30, 33, 34, 35, 36], but the delivery relative to the target was not reported in any study.

Protein targets and delivery were mentioned in only 10 studies [12, 17, 22, 24, 31, 33, 34, 35, 36]. Similar to energy targets, one study reported adhering to local policy without providing specific details [18]. Protein targets ranged from 1.0 to 1.1 g/kg/d across the studies, but there was no detail regarding the basis for selecting the weight used to determine these values (e.g. pre‐pregnancy, actual or adjusted body weight). Protein targets were reported in various formats, including grams of protein per kilogram per day [17, 24, 31, 33, 36] or grams of amino acids [12, 22, 35]. One study mentioned the use of Branched‐Chain Amino Acid (BCAA) free protein, however this was motivated by specific management guidelines related to maple syrup urine disease [34].

Protein delivery was widely variable, with reported values ranging from 57 to 100 g of protein/d or 42 to 85 g amino acids/kg/d.

### 4.2 Micronutrient Supplementation

Eight studies considered micronutrient supplementation [16, 24, 29, 31, 33, 34, 35, 36]. Four reported the use of multivitamins or trace elements, with limited detail provided regarding the dose or route by which these were administered [16, 24, 31, 34] (Table 3). Common micronutrients or electrolytes that were supplemented included magnesium (*n* = 5) [16, 20, 34, 35, 36], phosphate (*n* = 4) [20, 34, 35, 36], iron (*n* = 2) [16, 24] and sodium (*n* = 2) [20, 34].

### 4.3 Reported Weight Change

Weight change across the intervention period was documented in 20 studies [14, 15, 17, 18, 20, 21, 22, 23, 24, 25, 27, 29, 30, 31, 32, 33, 34, 35, 36] and four [14, 16, 19, 25] made no mention of weight, either pre‐pregnancy or during the artificial nutrition support (Table 4). Given the nature of the included studies (e.g. case series or case reports) some studies reported both weight gain and weight loss for individual patients. Weight gain of varying degrees from 1.8 kg to 13 kg was mentioned in 14 studies [12, 17, 18, 20, 21, 22, 23, 24, 27, 32, 33, 34, 36]. Ten studies reported between 0.5 and 11 kg weight loss with most being unclear as to whether this was during the period ANS or acute illness [12, 15, 17, 18, 20, 28, 29, 30, 31, 35].

**Table 4:** Summary of gestation period, reported maternal and foetal outcomes related to artificial nutrition support.

Paper	Gestation period	Gestational age when ANS commenced	Type of ANS	Reported outcomes following ANS		
				Maternal outcomes	Foetal outcomes	Other outcomes
Adami et al, 1992 [12]	Patient 1: 41 weeks Patient 2: 40 weeks Patients 3–11: mean 34 (SD 6) weeks	Patient 1: 12 week for 25 days and 28 weeks until delivery Patient 2: 19 weeks for 20 days and 32 weeks for 30 days Patients 3–11: mean 23 (SD 12.7) weeks	Parenteral	Patient 1: Bodyweight stabilised, and nutritional parameters (albumin and transferrin) returned normal range. Required caesarean Patient 2: Weight gain, and nutritional parameters (albumin and transferrin) returned normal range. Required caesarean Patients 3–11: 7 × home parenteral nutrition 6 × patients weight stabilised 4 × patients weight increased 1 × weight loss	Patient 1: normal body weight at birth Patient 2: normal body weight at birth Patients 3–11: 5 x small for gestational age 1 x premature birth All others normal body weight at birth	Not reported
Boyce, 1992 [13]	Patient 1: Term (exact gestation not reported) Patient 2: 39 weeks	Patient 1: 17 weeks Patient 2: ~19 weeks	Enteral	Patient 1: Persistent nausea and vomiting until birth Return to non‐pregnancy weight by delivery Patient 1: Nausea and vomiting several times per week Cessation of enteral feeding at ~21 weeks Increased weight	Patient 1: Term birth at normal body weight Patient 2: Term birth at normal body weight	Not reported
Christodoulou et al, 2008 [14]	Patient 1: 39 weeks Patient 2: 39 weeks	Patient 1: 14 weeks and 5 days Patient 2: 11 weeks and 5 days	Parenteral	Patient 1: resolution of nausea, vomiting and transaminases. Patient 2: resolution of nausea, vomiting. Post‐natal development of breast abscess	Patient 1: Term birth at normal body weight Patient 2: Term birth at normal body weight	Not reported
D'Orsi et al, 2023 [15]	36 weeks	Not reported	Parenteral	Elective c‐section weight loss to below pre‐pregnancy weightlack of breast milk requiring exclusive infant formula feeding	Healthy infant mild jaundice and 12.6% body weight loss at discharge (4 days following birth)	Not reported
Fedorka and Sullivan, 2004 [16]	34 weeks	10 weeks	Parenteral and enteral feeding	Right upper arm thrombosis Finger contractures Pre‐term labour Caesarean Breech Presentation	Healthy infant	Not reported
Galletta et al, 2022 [17]	25–40 weeks (mean 35.4 week)	Not reported	Mixed	Weight loss over hospitalisation (−1.7 (SD 2.3) kg) Caesarean delivery in 76.5% of patients	27.8% born with low birth weight 16.7% with very low birth weight	Not reported
Grooten et al, 2017 [18]	EN = 39 (IQR 38–40) SC = 39 (IQR 37–40)	9 (SD 3) weeks	Enteral	No difference in maternal weight gain, duration of hospital stay, readmission rate, nausea and vomiting symptoms or quality of life.	No difference in birthweight between groups	Nose‐throat irritation (26 participants) Obstruction (3 participants) Tube dislocation (23 participants)
Hsu et al, 1996 [19]	37–41 weeks (mean 39.7 SD 1.3 weeks)	7–14 weeks (mean 10.4 SD 2.8 weeks)	Enteral	Improved HG symptoms 6/7 x required home enteral feeding	6 x full term, healthy weight births 1 x foetal death (1 twin) in first trimester	1 x dislodgement of tube2 x clogged and/or dislodged
Laglenne and Mogensen, 2022 [20]	n/a	7 weeks27 weeks	Mixed	Successfully weaned off PN	1 foetal death 1 elective termination of pregnancy	Not reported
Maxwell et al, 2010 [21]	18–40 weeks	12.9 + /−5.4 weeks	Enteral	8/9 patients gained weight 2/9 reported nausea 2/9 infection 4/9 pain 1 patient required caesarean section	8/9 patients no foetal complications	3/9 tube leakage 3/9 tube migration or clogging 2/9 tubes replaced due to clogging
Mulherin et al, 2019 [22]	34 weeks	22 weeks	Parenteral	2 x hospital readmissions Hyperglycaemia requiring insulin worsening pulmonary status and maternal malnutrition	Small for gestational age (below 5th percentile) required NICU respiratory and nutrition support for 15 days	Not reported
O'Connor and Quinn, 1994 [23]	34 weeks	26 weeks	Parenteral	6 weeks post‐natal patient had regained pre‐pregnancy weight	Baby born in 10–20th centile for gestation with no evidence of biochemical abnormality	Unable to tolerate fine bore tube insertion due to retching
Okon and Hussein, 1996 [24]	36 weeks	28 weeks	Parenteral	7.3 kg weight gain	Infant born in good condition	Nil
Peled et al, 2014 [25]	38.7 + /‐ 2.1 in TPN group 39.2 + /‐ 2.8 in control group	Not reported	Parenteral	122/599 (20.4%) required PNHG/control group: Higher rates of gestational diabetes, preeclampsia and placental abruption HG + PN group: lower rate of preterm delivery and lower rate of labour induction	HG + PN group compared with control: higher birth weight, higher birth weight percentile and lower rate of < 10th percentile as well as lower rate of composite morbidity and NICU admission	Nil
Roem, 2002 [36]	27 weeks	12 weeks	Enteral	Hypomagnesaemia (0.69 mmol/L) Thiamine deficiency unsuccess home enteral feeding due to tube dislodgement and refusal for re‐insertionPersistent vomiting Emergency caesarean	Prematurity and foetal death in both twins	
Spiliopoulos, 2013 [27]	37 weeks	29 weeks	Parenteral	2 kg weight gain caesarean section	Healthy infant	Nil
Stokke et al, 2015 [28]	88% > 24 weeks	PN group: 8.4 (8.3‐9.1) weeks EN group: 8.0 (7.7‐8.6) weeks	Mixed	Weight loss more significant in EN compared with PN. Shorter length of gestation in EN compared with PN. Longer length of hospitalisation in EN group.	40 aborted pregnancies (27 terminated) 16 preterm births 25 SGA	58 enteral tube removals: 8 requests due to discomfort 46 clogging 4 forceful vomiting.1 case of pneumothorax (receiving PN) 2 CVC removals due to infection and 1 due to obstruction
Tanabe et al, 2022 [29]	Not reported	14 weeks and 1 day	Parenteral	Not reported	Not reported	Zinc deficiency due to lack of trace element preparation in PN
Vaisman et al, 2004 [30]	Not reported	6–13 weeks	Enteral	Weight loss in hospital: 0.9 to 5.1 kg (mean 2.2 SD 1.1) Complete cessation of vomiting and retching after 1–13 days (mean 5 SD 4 days)	Not reported	2 EN associated diarrhoea3 tube dislodged due to forceful vomiting1 tube blocked
Vanderwoude and Scholten, 1995 [31]	30–40 weeks	11.5 weeks	Parenteral – with 3 patients discharged on home PN	Marked improvement in self‐reported emotional wellbeing weight gain uniformly achieved 3 Home PN	2 infants delivered at full term with normal weights 1 induced at 30 weeks due to chorioamnionitis 1 voluntary interruption of pregnancy 2 awaiting delivery at time of publication	
Webber et al, 1993 [32]	32 weeks	25 weeks	Parenteral	2 months following pregnancy ‐ dysphagia for solids due to oesophageal stricture	Healthy baby delivered by elective caesarean section.	Not reported
Weinberg et al, 1982 [33]	36.5 weeks	35 weeks	Parenteral	Unsuccessful EN with ongoing vomiting requiring transition to PN	Infant was found to be small for date with body weight below the 10th percentile	Not reported
Wessel et al, 2015 [34]	37 weeks	36 weeks	Supplementary parenteral	Preeclampsia Chorioamnionitis and failure to progress with labour	Healthy female infant with normal newborn screen results	Not reported
Zhao et al, 2017 [35]	Not reported	11 weeks	Parenteral	Aggravated nausea and vomiting with fat emulsion Palpitation and sweating during PN infusion	Not Reported	Not reported

Abbreviations: ANS, artificial nutrition support; APGAR, Appearance, Pulse, Grimace, Activity, and Respiration score; BMI, body mass index; BP, blood pressure; CVC, central venous catheter; EN, enteral nutrition; HC, head circumference; HG, hyperemesis gravidarum; IV, intravenous; KG, kilograms; L, litre; LBW, low birth weight; NG, nasogastric; NICU, neonatal intensive care unit; PEG, percutaneous endoscopic gastrostomy; PN, parenteral nutrition; QOL, quality of life; SC, standard care; SGA, small for gestational age; VLBW, very low birth weight.

The duration of nutrition intervention ranged from 1 day to 5 months (Table 4). Notably, one study [12], following a 12‐week intervention, reported the largest weight loss of ‐18 kg, albeit in a patient with severe HG and complications of ansitory postcibal syndrome following a biliopancreatic diversion 2 months prior to pregnancy.

## 5 Maternal and Foetal Outcomes

Reported gestation periods and maternal and foetal outcomes are summarised in Table 4. Outcomes varied considerably across the included studies, and given that the majority were case reports or case series, with only one RCT, the findings should be interpreted with caution and no causal conclusions can be drawn regarding the impact of artificial nutrition support on maternal or foetal outcomes.

The single RCT investigating early EN plus standard care versus standard care alone in 116 patients admitted with HG between 5 and 20 weeks of gestation found no difference in the primary outcome of birth weight [18]. Notably, 70% of patients in the EN group reported at least one mechanical complication associated with the feeding tube, including nose and throat irritation, tube obstruction and dislocation. Additionally, quality of life measured using validated tools (Hospital Anxiety and Depression Scale, 36‐Item Short‐Form Health Survey and EuroQol Visual Analogue Scale) was lower in the EN group compared with standard care. These findings highlight the importance of considering patient experience and tolerability alongside clinical outcomes when evaluating artificial nutrition support in this population, and raise questions about whether EN is the most appropriate modality for HG management.

Beyond the RCT, the heterogeneity of outcome reporting across the remaining studies precludes meaningful synthesis. Full details of reported outcomes are presented in Table 4.

## 6 Discussion

To the best of our knowledge, this is the first scoping review exploring the published literature surrounding artificial nutrition support during acute illness and pregnancy. This review revealed that the research literature related to this topic is limited, with only 24 studies spanning a 42‐year period addressing the issue. This was not only an issue of low quantity but also low quality, with the majority of studies being individual case reports or case series in small numbers of people and only one RCT. Though a quality assessment of the literature was not relevant to the scoping review [9], the majority of studies were case reports or case series. Whilst providing valuable insight into the topic, case reports and case series are lower quality in the hierarchy of evidence [37]. A key finding of this scoping review was the lack of reporting and lack of strong evidence relating to the use of enteral nutrition or parenteral nutrition during acute illness in pregnancy. The lack of research may be compounded by the fact that pregnancy is often an exclusion criterion from many artificial nutrition support studies. This lack of research on the combined impact of acute illness and pregnancy on nutrient requirements, assessment, delivery and monitoring of artificial nutrition support is a major unmet need.

### 6.1 Indications for Artificial Nutrition Support

HG was the most common indication for artificial nutrition support across the included literature (*n* = 15 studies), and therefore represents the population from which the most clinical learning can be drawn. The predominant mode of nutrition support used across HG studies was PN, reflecting the clinical reality that persistent vomiting frequently precludes safe and tolerable EN. However, EN via nasogastric or nasojejunal tube was also reported in several studies, and the only RCT identified in this review, the MOTHER trial, directly compared early EN plus standard care against standard care alone and found no significant difference in birth weight, its primary outcome [18]. Importantly, it also reported that more than two thirds of patients receiving EN experienced at least one mechanical complication from the feeding tube. Whilst this is a single trial with an inherently selected population, these findings raise important questions for clinical practice, specifically, whether PN may be the more appropriate and better‐tolerated modality for patients with moderate‐to‐severe HG, and whether the threshold for initiating PN should be lower than is currently practised in many settings. Future clinical guidance for HG should consider these tolerability findings alongside clinical outcomes when recommending nutrition support modality.

Data from a large, retrospective observational study provides some support for the benefit of timely nutritional intervention in HG, reporting higher birth weight and lower rates of composite neonatal morbidity in those receiving PN compared with controls [25]. Whilst this is observational data and subject to confounding, it reinforces the importance of ensuring nutritional adequacy in this group.

Although only case reports described artificial nutrition support in pregnant patients with CF, this population warrants specific discussion given the unique and compounding nutritional challenges it presents. CF is associated with malabsorption, pancreatic exocrine insufficiency, and chronically elevated energy expenditure [26], all of which are further compounded by the metabolic demands of pregnancy and the nutritional impact of acute pulmonary exacerbations. Both CF case reports used PN, and both reported difficulty achieving adequate nutritional targets. The first described a case requiring 85 days of PN with energy targets progressively escalated up to 61 kcal/kg/day in an attempt to achieve target gestational weight gain, yet the infant was born small for gestational age [22]. This suggests that standard energy targets derived from healthy pregnancy guidance may be substantially insufficient for pregnant patients with CF, and that these patients may require significantly higher energy provision than would typically be anticipated. Clinicians managing pregnant patients with CF should anticipate early and intensive nutritional intervention, with frequent reassessment of targets in response to weight trajectory and clinical status. The advent of highly effective Cystic Fibrosis Transmembrane Conductance Regulator modulator therapies, which improve nutritional status and lung function in CF [26], may alter the nutritional landscape of CF in pregnancy and as such, prospective reporting of nutrition support practices in this evolving population is therefore a priority for future research.

### 6.2 Variability in Estimation of Nutritional Requirements and Nutrient Provision

Provision of adequate energy and protein throughout pregnancy is paramount for supporting intrauterine growth and underfeeding and overfeeding of both energy and protein may lead to suboptimal outcomes in line with other acutely unwell populations. Current guidance for healthy pregnancies suggests that energy and protein requirements are dependent on gestation. However, we did not identify any studies investigating energy or protein requirements in the acutely ill pregnant population. The lack of robust evidence may explain the variation in practice across the studies included in this scoping review. The gold standard for determining energy expenditure in acute illness is indirect calorimetry (IC) [38], however, this is not routinely used in clinical practice and means that the reliance on predictive equations and consistent monitoring should be a cornerstone of nutritional management of hospitalised patients who are pregnant [39]. However, only a limited number of studies reported the method of calculating estimated energy requirements. Within this review, the most commonly used equations to estimate energy requirements were the IOM predictive equation for resting energy expenditure (REE), Harris Benedict equation and various other weight‐based equations, with some studies adding additional energy to account for the gestation period. This variation in practice is a reflection of the limited evidence and consensus in this area. Given the lack of evidence, a new predictive equation has been developed, however, this has not been validated in pregnancy in acute illness and further work is needed before it could be recommended for use [37].

Interestingly, fewer studies reported estimated protein requirements (targets) than energy targets which may indicate that either more importance is placed on energy provision during artificial nutrition support in the acutely unwell pregnant patient or it may reflect the limited evidence available to support targets. Where protein targets were reported they were between 1.0 and 1.1 g/kg/d, in line with current recommendations of 0.8–1.1 g/kg/day. However, a recent study has challenged this guidance and based on the Indicator Amino Acid Oxidation method, suggested that protein requirements may indeed be higher, at 1.2 g/kg/d at around 16 weeks gestation and 1.6 g/kg/d at around 36 weeks' gestation [40]. However, that study was conducted in healthy pregnancy and did not consider the metabolic impact of acute illness or additional losses. These findings clearly indicate the need for further research focussed on hospitalised pregnant patients.

This review highlights a significant gap in the reporting and monitoring of nutritional adequacy. Energy and protein prescription practices varied considerably across studies, and delivery relative to target was rarely reported, making it impossible to determine whether patients received adequate nutrition during their admission. This finding is consistent with the broader EN and PN literature where reporting of RCTs investigating enteral or parenteral nutrition interventions in critical illness has been demonstrated to be frequently incomplete even when assessed against the CONSORT statement, and proposed minimum standard reporting criteria to address this gap [41], criteria which the majority of studies identified in this review would be unlikely to meet.

Whilst the impact of poor nutritional delivery in the acutely unwell pregnant population is unknown, it is likely that this will also have implications for both maternal and foetal outcomes, especially if this is prolonged. Indeed, one observational study included in this review reported favourable outcomes with early artificial nutrition support contributing to positive foetal outcomes such as higher birthweight [25]. Understanding nutritional adequacy may be particularly important in condition such as HG which can be prolonged, in some cases persisting across the majority of the pregnancy and the consequences of sustained nutritional inadequacy may be clinically significant for both mother and foetus.

Given the lack of evidence guiding energy and protein targets in this population, close monitoring is essential and this should include regular review of the delivery of energy and protein and nutritional status so that feeding targets can be adjusted accordingly.

### 6.3 Micronutrient Supplementation

Current UK guidelines recommend supplementing iron, folate, calcium and vitamin D during pregnancy in healthy women [42], however, only six studies reported supplementing additional micronutrients in acute illness. There are several reasons that may explain this low number. First, in hospital settings, supplementation is usually based on low serum levels and yet micronutrient levels are not routinely measured due to the requirement for specialist laboratory facilities and because the results being impacted by inflammation [39] and. It is possible that without data for serum levels, there was low confidence to supplement. Second, guidelines for micronutrient supplementation differ between countries and 11 countries are represented within this review. Third, enteral formulas generally provide all micronutrients, potentially explaining the limited reporting of additional micronutrient supplementation. However, this assumption relies on the complete delivery of EN which does not routinely occur in practice [43]. Finally, most studies are case series with different aims (e.g. reporting on medical management of HG) and therefore micronutrient supplementation may have been provided but not reported.

### 6.4 Weight Changes Across Pregnancy

This scoping review identified a notable gap in the routine reporting of maternal weight prior to pregnancy and during the artificial nutrition support intervention. Despite ongoing discussions regarding the use of weight as a tool for monitoring nutritional interventions, it is still considered standard practice across a range of clinical settings [44]. There is consistent reporting amongst research of the importance of gestational weight gain in predicting foetal and maternal outcomes [45], hence, understanding what these parameters are is key for clinicians, especially in the context of providing artificial nutrition support in the acute setting. At present, guidelines from the IOM suggest targets for weight gain across pregnancy should be individualised and relative to the pre‐pregnancy BMI, this is recommended to prevent excessive gestational weight gain and postpartum retention [45]. Given that BMI was not consistently reported, it is difficult in the scope of this review to comment on gestational weight gain and the effectiveness of the artificial nutrition support interventions provided.

Despite nutritional screening, which includes measuring weight, being mandatory in many countries, some clinical environments and conditions pose barriers to weighing patients (e.g. in unstable critically ill patients, or in patients too unwell to mobilise). Interestingly, a cross sectional survey of 149 pregnant patients found that mid upper arm circumference (MUAC) and subscapular skinfolds (SBSF) exhibited greater correlations with maternal visceral and total adipose tissue than pre‐pregnancy BMI [46]. Alarming, none of these methods of measuring anthropometry beyond body weight were reported in any of the studies of artificial nutrition support in this scoping review. These findings could prove of benefit as proxy measures in clinical practice and should be explored further.

### 6.5 Maternal and Foetal Outcomes

There was considerable heterogeneity in the outcomes reported in the studies in this scoping review and given that the majority of studies reviewed were case reports and case series, we are not able to determine any causation between the use of artificial nutrition support and the reported maternal and foetal outcomes.

### 6.6 Strengths and Limitations

A key strength of this scoping review is that it is the first of its kind to identify and describe the literature surrounding artificial nutrition support acute illness in pregnancy. Through doing so, it is possible to examine gaps within the literature and enhance provision of healthcare to promote positive outcomes in this cohort. This acts as a stepping stone in ensuring care can be standardised and pregnant patients can be represented in the literature.

The limitations of this review must be acknowledged. In terms of the scoping review methodology, the exclusion of papers not written in English language may have limited the range of results obtained. Meanwhile the final search date (March 2024), means that additional eligible reports may have subsequently been published, however, with very few studies relevant to this research question each year (on average 0.6 papers per year), this is unlikely to constitute a major body of additional evidence. In terms of the limitations of the source data identified, the fact that the majority of the literature are case studies or case series means the overall quality of the data presented is poor, and prevents recommendations for application to practice from being made.

## 7 Conclusion

This scoping review is the first to map the published literature on artificial nutrition support during acute illness in pregnancy. The evidence base is limited in both quantity and quality, comprising predominantly case reports and case series spanning 42 years, with only one RCT. Considerable heterogeneity in practice and a lack of standardised outcome reporting were identified, limiting evidence synthesis. Ethical and practical barriers to including pregnant patients in nutritional research mean that high‐quality trials in this population are unlikely, making robust observational and audit data all the more critical. The findings from this review suggest that future research and clinical audit in acutely unwell pregnant patients should prioritise standardised reporting of energy and protein targets and delivery, documentation of the rationale for nutrition support modality selection, and systematic recording of complications and patient‐reported outcomes. The development of clinical guidelines specific to this population remains an important unmet need.

## Author Contributions

**Danielle E. Bear:** conceptualisation, methodology, data curation, investigation, supervision, writing – review and editing. **Hanna Tejani:** methodology, data curation, investigation, writing – original draft preparation. **Ali Morrison:** data curation, investigation, writing – review and editing. **Kevin Whelan:** conceptualisation, methodology, supervision, writing – review and editing.

## Funding

The authors have nothing to report.

## Supporting information


Supporting File 1

Supporting File 2

## Data Availability

The data that support the findings of this study are available from the corresponding author upon reasonable request.
