# Supplementary material for: Artificial Nutrition Support During Acute Illness in Pregnancy: A Scoping Review
Source: J Hum Nutr Diet. 2026 Jul 20;39(4):e70315. doi: 10.1111/jhn.70315 (PMC13385647; doi:10.1111/jhn.70315)
Supplement: Supplementary file 2 — Supporting File 2 [file JHN-39-0-s002.pdf]

## Supplementary Appendix 1

**Search Strategy (MEDLINE)**
All searches were based off MEDLINE search strategy

| Search | Query |
|---|---|
| 1 | exp Nutritional Support/ or nutrition* support.mp |
| 2 | exp Nutrition Therapy/ or nutrition* management.mp. |
| 3 | enteral feeding.mp. or exp Enteral Nutrition/ |
| 4 | parenteral nutrition/ or parenteral nutrition, total/ |
| 5 | nutrition* intervention.mp. [mp=title, book title, abstract, original title, name of substance word, subject heading word, floating sub-heading word, keyword heading word, organism supplementary concept word, protocol supplementary concept word, rare disease supplementary concept word, unique identifier, synonyms, population supplementary concept word, anatomy supplementary concept word] |
| 6 | nutrition*.mp. [mp=title, book title, abstract, original title, name of substance word, subject heading word, floating sub-heading word, keyword heading word, organism supplementary concept word, protocol supplementary concept word, rare disease supplementary concept word, unique identifier, synonyms, population supplementary concept word, anatomy supplementary concept word] |
| 7 | exp Vitamins/ or exp Micronutrients/ or exp Dietary Supplements/ or micronutrient supplement*.mp. |
| 8 | hospital*.mp. or exp Hospital Units/ |
| 9 | exp Critical Illness/ or exp Intensive Care Units/ or critical* ill*.mp. |
| 10 | critical care.mp. or exp Critical Care/ |
| 11 | ((intensive or critical) adj1 care).mp. [mp=title, book title, abstract, original title, name of substance word, subject heading word, floating sub-heading word, keyword heading word, organism supplementary concept word, protocol supplementary concept word, rare disease supplementary concept word, unique identifier, synonyms, population supplementary concept word, anatomy supplementary concept word] |
| 12 | ((intensive or critical) adj1 care unit).mp. [mp=title, book title, abstract, original title, name of substance word, subject heading word, floating sub-heading word, keyword heading word, organism supplementary concept word, protocol supplementary concept word, rare disease supplementary concept word, unique identifier, synonyms, population supplementary concept word, anatomy supplementary concept word] |
| 13 | exp Hospitalization/ or hospitali#*.mp |
| 14 | acute* ill*.mp. [mp=title, book title, abstract, original title, name of substance word, subject heading word, floating sub-heading word, keyword heading word, organism supplementary concept word, protocol supplementary concept word, rare disease supplementary concept word, unique identifier, synonyms, population supplementary concept word, anatomy supplementary concept word] |
| 15 | exp peripartum period/ or pregnancy/ or exp pregnancy outcome/ |
| 16 | pregnan*.mp. [mp=title, book title, abstract, original title, name of substance word, subject heading word, floating sub-heading word, keyword heading word, organism supplementary concept word, protocol supplementary concept |

| | |
|---|---|
| | word, rare disease supplementary concept word, unique identifier, synonyms, population supplementary concept word, anatomy supplementary concept word] |
| 17 | obstetric*.mp. |
| 18 | 1 or 2 or 3 or 4 or 5 or 6 or 7 |
| 19 | 8 or 9 or 10 or 11 or 12 or 13 or 14 |
| 20 | 15 or 16 or 17 |
| 21 | 18 and 19 and 20 |
